# Impact of Spaceflight and Artificial Gravity on the Mouse Retina: Biochemical and Proteomic Analysis

**DOI:** 10.3390/ijms19092546

**Published:** 2018-08-28

**Authors:** Xiao W. Mao, Stephanie Byrum, Nina C. Nishiyama, Michael J. Pecaut, Vijayalakshmi Sridharan, Marjan Boerma, Alan J. Tackett, Dai Shiba, Masaki Shirakawa, Satoru Takahashi, Michael D. Delp

**Affiliations:** 1 Department of Basic Sciences, Division of Biomedical Engineering Sciences (BMES), Loma Linda University School of Medicine and Medical Center, Loma Linda, CA 92350, USA; nnishiyama@llu.edu (N.C.N.); mpecaut@llu.edu (M.J.P.); 2 Department of Biochemistry and Molecular Biology, University of Arkansas for Medical Sciences, Little Rock, AR 72205, USA.; sbyrum@uams.edu (S.B.); ajtackett@uams.edu (A.J.T.); 3 Arkansas Children’s Research Institute, Little Rock, AR 72202, USA; 4 Division of Radiation Health, Department of Pharmaceutical Sciences, University of Arkansas for Medical Sciences, Little Rock, AR 72205, USA; vmohanseenivasan@uams.edu (V.S.); mboerma@uams.edu (M.B.); 5 JEM Utilization Center, Human Spaceflight Technology Directorate, JAXA, Tsukuba 305-8505, Japan; shiba.dai@jaxa.jp (D.S.); shirakawa.masaki@jaxa.jp (M.S.); 6 Department of Anatomy and Embryology, University of Tsukuba, Tsukuba 305-8575, Japan; satoruta@md.tsukuba.ac.jp; 7 Department of Nutrition, Food and Exercise Sciences, Florida State University, Tallahassee, FL 32306, USA; mdelp@fsu.edu

**Keywords:** spaceflight, ocular tissue, microgravity, artificial gravity, apoptosis, proteomics, oxidative stress

## Abstract

Astronauts are reported to have experienced some impairment in visual acuity during their mission on the International Space Station (ISS) and after they returned to Earth. There is emerging evidence that changes in vision may involve alterations in ocular structure and function. To investigate possible mechanisms, changes in protein expression profiles and oxidative stress-associated apoptosis were examined in mouse ocular tissue after spaceflight. Nine-week-old male C57BL/6 mice (*n* = 12) were launched from the Kennedy Space Center on a SpaceX rocket to the ISS for a 35-day mission. The animals were housed in the mouse Habitat Cage Unit (HCU) in the Japan Aerospace Exploration Agency (JAXA) “Kibo” facility on the ISS. The flight mice lived either under an ambient microgravity condition (µg) or in a centrifugal habitat unit that produced 1 *g* artificial gravity (µg + 1 *g*). Habitat control (HC) and vivarium control mice lived on Earth in HCUs or normal vivarium cages, respectively. Quantitative assessment of ocular tissue demonstrated that the µg group induced significant apoptosis in the retina vascular endothelial cells compared to all other groups (*p* < 0.05) that was 64% greater than that in the HC group. Proteomic analysis showed that many key pathways responsible for cell death, cell repair, inflammation, and metabolic stress were significantly altered in µg mice compared to HC animals. Additionally, there were more significant changes in regulated protein expression in the µg group relative to that in the µg + 1 *g* group. These data provide evidence that spaceflight induces retinal apoptosis of vascular endothelial cells and changes in retinal protein expression related to cellular structure, immune response and metabolic function, and that artificial gravity (AG) provides some protection against these changes. These retinal cellular responses may affect blood–retinal barrier (BRB) integrity, visual acuity, and impact the potential risk of developing late retinal degeneration.

## 1. Introduction

Approximately 30% of astronauts on short-term (~2 week) Space Shuttle flights and 60% on long-duration (~6 month) missions to the International Space Station (ISS) are reported to have experienced some impairment in distant or near visual acuity [1]. Previous studies of rodents also show that environmental conditions associated with spaceflight and simulated microgravity induce alterations in retinal structure and function [2,3,4].

Retinal damage and degeneration can occur as a result of multiple factors, including aging, ischemia, fluctuation in oxygen tension, oxidative stress, and increased intraocular pressure. Our previous data from Space Shuttle studies showed a significant increase of apoptosis in retinal cells of flight mice compared to ground controls [2], which could lead to morphologic changes and impairment of retinal function. However, the pathophysiological consequences and cellular mechanisms of stress stimuli, especially those associated with a spaceflight environment, in facilitating retinal damage are less studied and remain unclear.

Data on the response of biochemical systems to altered gravity is very limited in animal studies [5]. Some lines of evidence suggest that one of the mechanisms involved in response to spaceflight, including changes in the gravity vector, is likely due to oxidative stress [2,6,7]. Studies have shown that exposure to microgravity during spaceflights is associated with elevations in oxidative stress and production of lipid peroxidation in both humans and rodents [8,9]. However, our knowledge about the duration or dose of microgravity exposure needed to induce oxidative stress or alterations in retinal structure and function is extremely limited. For example, one study has shown that even transient exposure to microgravity produced by parabolic flight can cause alterations in retinal vasculature to occur [1]. One means to address the impact of microgravity conditions is through the application of artificial gravity (AG) during spaceflight.

Creating AG by centrifugal force makes it possible to have a partial gravity or Earth-like gravity (1 *g*) control in space, where all other environmental conditions associated with spaceflight are present. A newly developed mouse habitat cage unit (HCU) by Japan Aerospace Exploration Agency (JAXA) has been installed in the Centrifuge-equipped Biological Experiment Facility (CBEF) on board the ISS [10]. This rodent centrifugation capability provides opportunities to compare the effectiveness of AG prescription under spaceflight conditions. Since the HCU can reproduce Earth-like gravity, it can be used to test the effectiveness of AG to mitigate the effects of microgravity on physiological systems [11].

The goal of the present study was to investigate the effects of spaceflight on oxidative stress and apoptosis in retinal endothelial cells and to identify spaceflight-induced changes in protein expression profiles in mouse ocular tissue. Additionally, we sought to determine whether the application of 1 *g* AG during spaceflight could mitigate any detrimental effects of microgravity on the retina. We hypothesized that spaceflight would induce elevations in oxidative stress and apoptosis in retinal endothelial cells, as well as alter ocular proteins associated with apoptosis, cell repair, inflammation and metabolic function. We further hypothesized that the application of 1 *g* AG would mitigate these changes.

## 2. Results

### 2.1. Apoptosis in Retinal Endothelial Cells Following Spaceflight

**Figure 1:**
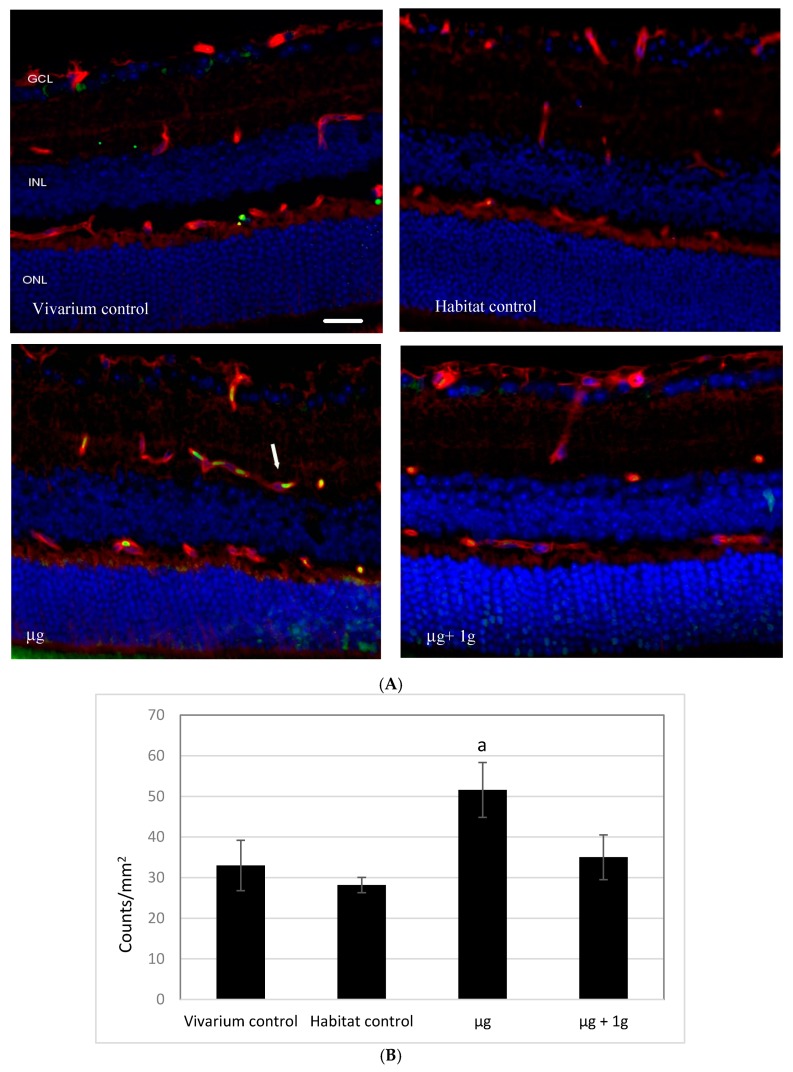

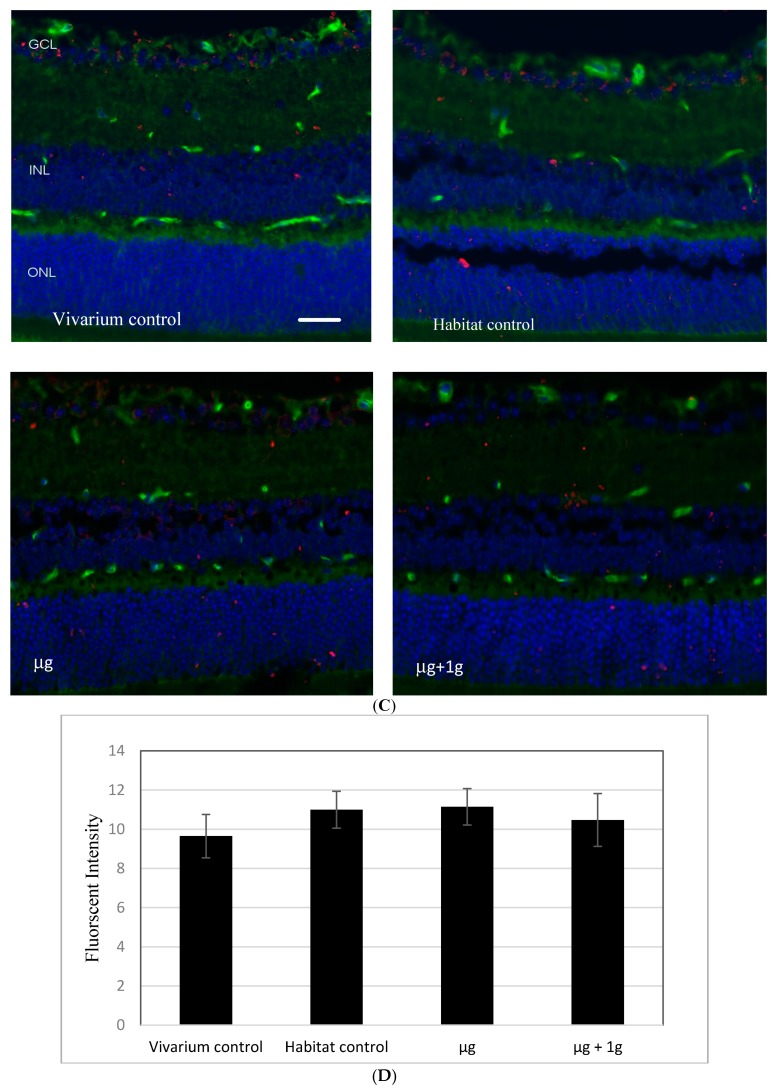
(**A**) Apoptosis based on terminal deoxynucleotidyltransferase dUTP nick-end labeling (TUNEL) staining of 9-week old male C57BL/6 mouse retinal tissue. Groups (*n* = 6): Vivarium control, habitat control, µg, and µg + 1 *g*. TUNEL-positive cells were identified with green fluorescence, the endothelium was stained with lectin (red). The nuclei of photoreceptors were counterstained with DAPI (blue). In the control retinal tissue, only sparse TUNEL-positive cells were found. In the retina from µg mice, TUNEL-positive labeling was apparent in the retinal endothelial cells. Arrow: TUNEL-positive endothelial cell. Outer nuclear layer (ONL); inner nuclear layer (INL); ganglion cell layer (GCL). Scale bar = 50 µm; (**B**) immunoreactivity of TUNEL staining in the retinal endothelium. Values are represented as mean density ± standard error of the mean (SEM) for a 6 mice/group, and the density profiles were expressed as mean number of apoptotic positive cells/mm^2^. The mean of the density profile measurements across 5 retina sections per eye was used as a single experimental value. ^a^ significantly higher than all other groups (*p* < 0.05); (**C**) immunoreactivity of 4-HNE staining in the 9-week old male C57BL/6 mouse retina. 4-HNE positive staining was identified with red fluorescence; the nuclei were counterstained with 4′,6-diamidino-2-phenylindole (DAPI, blue). The vessel was stained with tomato lectin (green). Scale bar = 50 µm; (**D**) the averages fluorescence intensity for 4-HNE activity were measured and calculated using the ImageJ program. Fluorescence was averaged across 5 retinas per group. Values are represented with mean + SEM. No significant differences among groups were detected.

Immunocytochemical analysis by terminal deoxynucleotidyltransferase dUTP nick-end labeling (TUNEL) assay showed that spaceflight conditions induced significant apoptosis in the retinal endothelial cells (Figure 1A) relative to that in the habitat and vivarium control conditions and in the AG (µg + 1 *g*) condition. Our quantitative assessment revealed that the density of apoptotic cells in the retina was the highest in the µg group, and was 64% greater than that in the habitat control group (Figure 1B).

### 2.2. 4-Hydroxynonenal (4-HNE) Immunoreactivity Following Spaceflight

Reactive oxygen species (ROS) are involved in lipid peroxidation and membrane lipids are among the major targets of ROS. The occurrence of lipid peroxidation was evaluated with immunohistochemistry with an antibody against 4-HNE, which is an indicative marker of oxidative damage to the retina (Figure 1C). There were no significant differences among groups in the level of 4-HNE immunoreactivities (Figure 1D). 

### 2.3. Proteomics on Mouse Ocular Tissue

**Figure 2:**
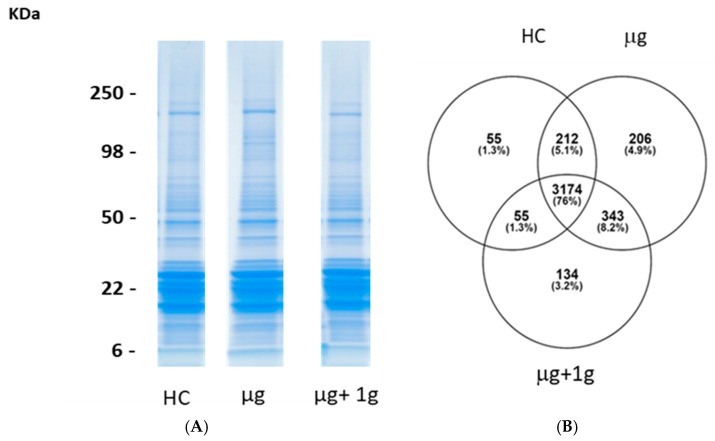
Proteins identified from µg, μg + 1 *g*, and habitat controls (HC) by high-resolution mass spectrometry. (**A**) Five micrograms of protein was resolved by one-dimensional sodium dodecyl sulfate–polyacrylamide gel electrophoresis (SDS-PAGE) and visualized by Coomassie-staining. Eyecups contain retinal layers were used for proteomic analysis. Samples were analyzed with six biological replicates. Each gel lane was sliced into 24 equivalent sections. Protein in each gel slice was digested in-gel with trypsin and identified by high-resolution mass spectrometry; (**B**) a Venn diagram represents the number of total proteins identified from each group. Seventy-six percent of the proteins were identified in all three groups.

Protein expression profile analysis were focused on comparisons between flight groups vs habitat control (HC). Analyzing the proteomic changes induced by flight conditions vs the HC group is more relevant for determining the effects of weightlessness and AG since HC mice were placed in the same flight hardware (cages) used in flight and environmental parameters such as temperature, humidity and carbon dioxide (CO_2_) levels were matched to that during spaceflight. Five micrograms of protein from each eyecup sample was resolved by 4–20% sodium dodecyl sulfate (SDS) Tris-Gly gel electrophoresis, visualized by Coomassie stain, in-gel trypsin digested, and analyzed by LC/MS on an LTQ Orbitrap Velos mass spectrometer. Figure 2A shows a gel image depicting one representative sample for each of the three sample groups. 

A MaxQuant database search (restricted to Mus musculus) identified a total of 4179 proteins from all 18 samples. A Venn diagram indicates that we identified 3174 (76%) common proteins in all groups and, therefore, differences between the groups are reflective of the intensities of the proteins detected (Figure 2B) [12]. A small percentage of proteins were uniquely identified in each of the µg + 1 *g*, µg, and HC samples and may reflect specific changes within each group. The MS1 precursor intensities were converted to relative iBAQ intensities by dividing the iBAQ intensity by the sum of all iBAQ intensities in the sample [13]. The samples were normalized based on the median relative iBAQ intensities for each sample, log_2_ transformed, and missing values were imputed based on the normal distribution. The log_2_ normalized iBAQ intensities were used for analyses.

**Figure 3:**
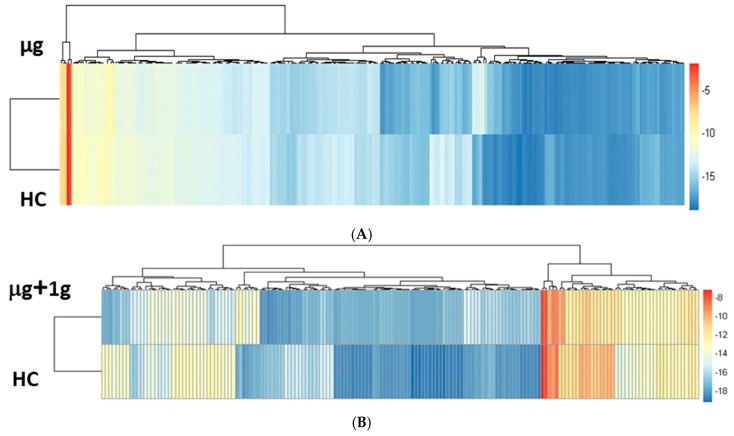
Unsupervised hierarchical clustering of significantly differentiating proteins. (**A**) Significant proteins between the µg versus habitat controls (HC). 250 Significant proteins based on false discovery rate (FDR) adjusted (*p* < 0.05); (**B**) significant proteins between µg + 1 *g* versus HC. 171 Significant proteins based on FDR adjusted (*p* < 0.05). Unsupervised hierarchical clustering of the log_2_ normalized iBAQ intensities for significantly differentiating proteins was performed using the Euclidean distance metric with oheatmap R package. The hierarchical cluster was generated for comparison and visually represents the significant protein intensities for each sample group. The intensities were standardized by the mean and standard deviation before clustering. Proteins were considered significant based on Mann–Whitney U FDR corrected *p*-value < 0.05.

**Figure 4:**
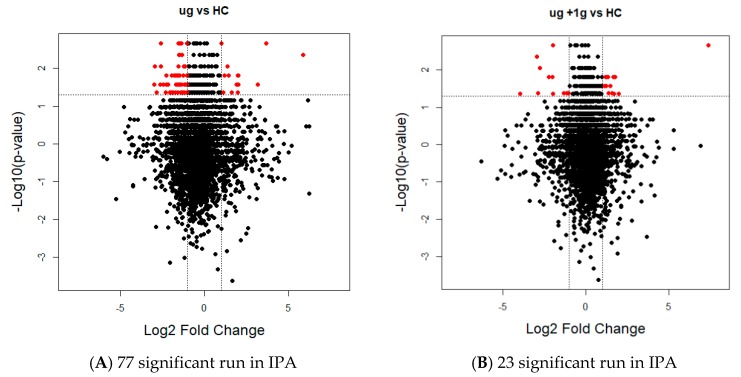
Volcano plots show differentially expressed proteins (FDR adjusted *p* < 0.05 and fold change >2). (**A**) µg versus HC: 77 significant run in Ingenuity Pathway Analysis (IPA); (**B**) µg + 1 *g* versus HC: 23 significant run in IPA. Volcano plots were generated based on fold-change of protein levels using the log_2_ normalized iBAQ intensities from six biological replicates. The x-axis indicates a log_2_ fold-change and the *y*-axis indicates −log_10_ *p*-value based on a Mann–Whitney U test with a FDR adjusted *p*-value. The horizontal line indicates a *p*-value < 0.05 and the vertical lines represent a fold-change >2.

We identified 250 and 171 significantly differentiating proteins comparing the µg versus HC and µg + 1 *g* versus HC sample groups, respectively, based on a Mann–Whitney U test with a false discovery rate (FDR) corrected *p*-value < 0.05. A hierarchical cluster was generated for both comparisons (Figure 3) and visually representing the significant protein intensities for each sample group. We further filtered the list of significant proteins by using a fold change >2 threshold. By using a *p*-value and a protein expression threshold, we were able to identify the top significant proteins. These top significant proteins are seen in the heat maps as the clusters of proteins with the largest changes in intensity (yellow to blue) (Figure 3). Volcano plots were used to visualize all identified proteins and highlight the significant proteins for each comparison in the study using R studio (Figure 4). The y-axis consists of −log_10_ *p*-values based on the Mann–Whitney U FDR adjusted *p*-values, while the *x*-axis consists of the log_2_ fold change. The vertical lines indicate up- and down expression using a fold change >2 threshold. The horizontal line indicates a *p*-value of 0.05. We identified 77 and 23 significant proteins based on these two criteria for the µg versus HC and µg + 1 *g* versus HC, respectively.

**Figure 5:**
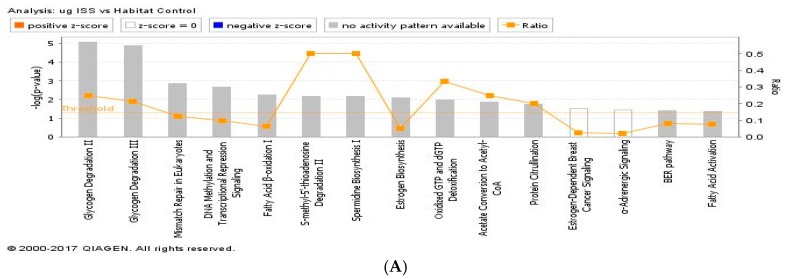

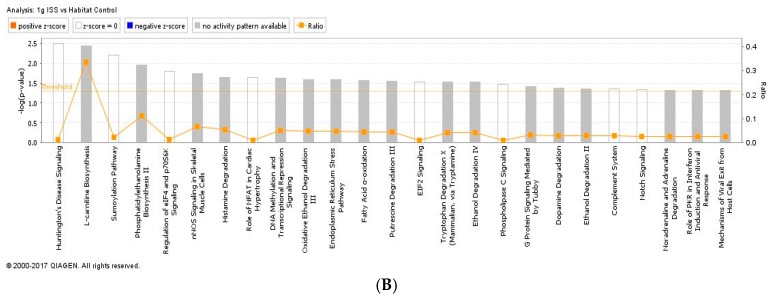
(**A**) IPA of top canonical pathways altered between µg and habitat control (HC) ocular tissue; (**B**) IPA of top canonical pathways altered between µg + 1 *g* and HC ocular tissue. −log_10_ (*p*-value) ≥ 1.3 = significant at *p* ≤ 0.05. The differentially expressed proteins were analyzed by IPA.

These differentially expressed proteins were then analyzed using Ingenuity Pathway Analysis (IPA, QIAGEN Redwood City, www.qiagen.com/ingenuity) [14]. There were significant changes in canonical pathways and upstream regulators in the pathway analysis for flight groups compared to the HC controls. Our proteomic analysis identified only 33 upstream regulator proteins based on the Z-scores used to predict either activation (Z-score > 0) or inhibition (Z-score < 0) in the µg + 1 *g* animals compared to HC mice that involves disease development, molecular/cellular function and cell signaling, while 60 regulators were identified in the µg group compared to HC mice. IPA analysis revealed many key pathways are affected that are responsible for cell death, cell repair, inflammation, carbohydrate metabolism, mitochondrial function, fatty acid metabolism, and oxidative phosphorylation when comparing the µg group with HC group. Pathways were considered significant based on the Fisher exact test with a −log_10_ *p*-value > 1.3 (corresponds to a *p*-value < 0.05). Significantly affected canonical pathways between µg + 1 *g* vs. HC or µg group vs HC or are shown in Figure 5A,B, respectively. There were also significant changes in regulated protein expression in the pathway analysis for µg or µg + 1 *g* groups compared to HC group or µg group vs. µg + 1 *g* group. There was very little overlap of significantly regulated protein between µg vs. HC and µg + 1 *g* vs. HC. In the µg group, there was significant alteration in functions related to overall organismal survival, cellular assembly and metabolism. Significant increased or decreased protein expression levels are summarized and listed in Table 1, Table 2 and Table 3.

## 3. Discussion

The current study demonstrates that spaceflight induces apoptosis in retinal vascular endothelial cells. We also identified spaceflight-induced changes in proteomic profiles and pathways in the ocular tissue. The results indicate that spaceflight induces changes in neuronal structure, cellular organization, mitochondrial function and oxidative phosphorylation and inflammation which, in turn, may lead to tissue injury and late neurodegeneration. This study is the first to investigate the role of AG provided by centrifugation during spaceflight as a countermeasure for mitigating putative effects of microgravity on ocular structure and function. 

In our study, several oxidative stress-related signaling pathways are significantly altered in the µg group, which includes fatty acid β-oxidation I, fatty acid activation and α-adrenergic pathway. However, we did not find a significant change in an oxidative stress marker for lipid peroxidation, 4-HNE when measured two days after splash-down. In our previous space shuttle Atlantis (STS-135) study, the level of HNE protein was significantly elevated in the retina after spaceflight compared to controls [2]. This is not surprising as these two flight conditions are different. Female C57BL/6 mice were flown in the STS-135 for 13-day mission, and within 3–5 h of landing, eyes were removed for analysis. In the current study, male mice were flown to the ISS for a 35-day mission, and the mice were euthanized at about 40 h after splashing down. Differences in gender, mission duration and recovery time might contribute to their dynamic stress response. The level of oxidative stress can be affected by their stress reaction to a prolonged flight in space and in the period of re-adaptation to the normal gravity after landing [15]. Another possible explanation for the lack of significant change in 4-HNE in the current study is that spaceflight might elicit protective effects in the central nervous system (CNS) by inhibiting some gravity-initiated stress signaling pathway.

**Figure 6:**
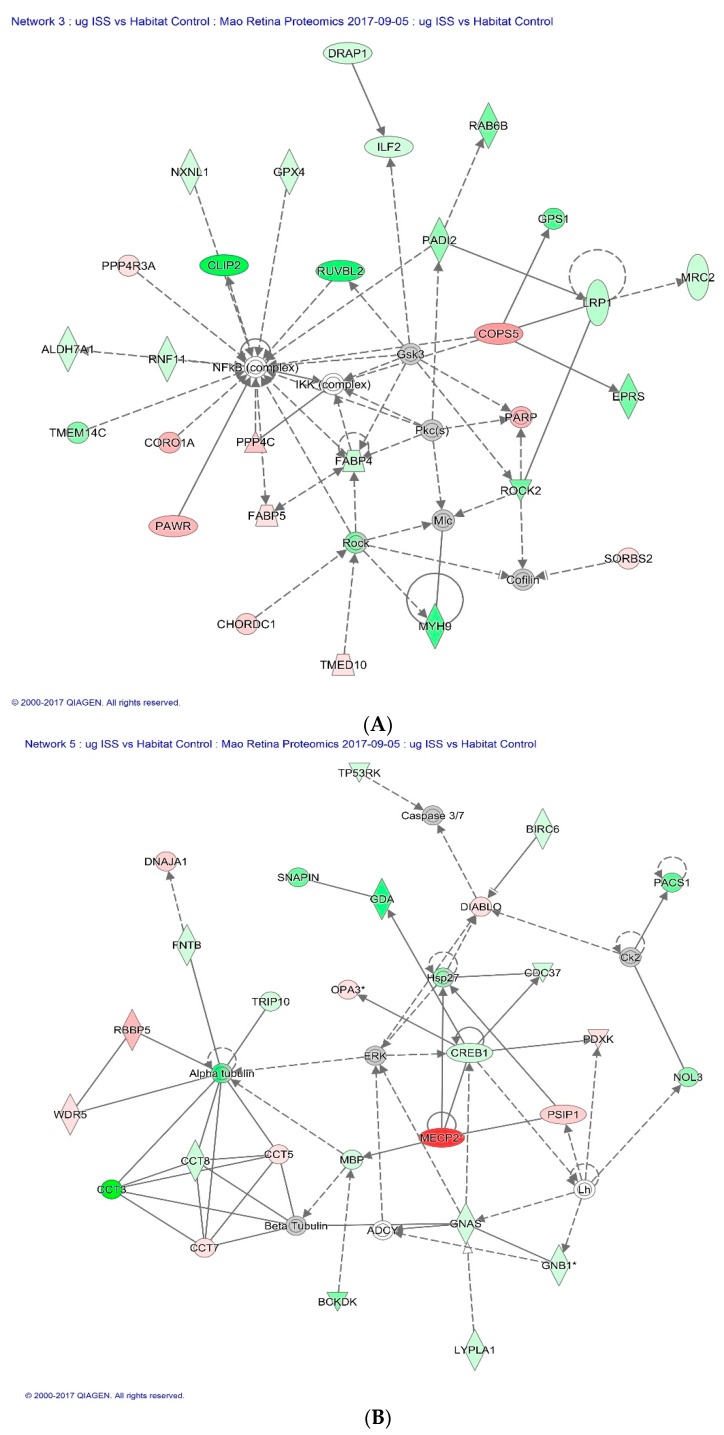
(**A**) Impact of spaceflight condition (µg group) on CLIP2 expression in the network of proteins responsible for cellular assembly and organization, cell signaling and interaction. Red = up-regulated (*p* < 0.05). Grey = un-changed. Green = down-regulated (*p* < 0.05); (**B**) Impact of spaceflight condition (µg group) on MECP2 protein expression in the network of proteins involving in cell death and survival response. Red = up-regulated (*p* < 0.05). Grey = un-changed. Green = down-regulated. (**A**,**B**) are generated with IPA software. More detailed description of the legends (squares, circles, etc.) can be found at the Ingenuity Systems website.

Of all the proteins significantly altered by spaceflight, the Cap-Gly domain containing linker protein 2 (CLIP2), also known as CLIP-115, was significantly down-regulated in both µg and µg + 1 *g* groups. This protein is found predominantly in the CNS, where it likely plays a role in the normal structure and function of nerve cells. Within cells, this protein is thought to regulate cell cytoskeleton function that helps to determine cell shape, size, and movement [16,17,18,19,20]. Figure 6A illustrates CLIP2 in the network of proteins responsible for cellular assembly and organization, cell signaling and interaction as defined by IPA in response to spaceflight.

Methyl-CpG-binding protein 2 (MECP2) had the highest fold changes in flight mice (µg group) compared to HC. This protein binds to methylated DNA and regulates chromatin structure and transcription [21,22]. MECP2 is most abundantly produced in the brain where it is expressed primarily in post-mitotic neurons [23]. Previous work has also shown MECP2 mediated epigenetic regulation of senescent endothelial progenitor cells and promoted apoptosis [24]. Furthermore, a recent study provides evidence that elevated MECP2 in mice causes neurodegeneration [25]. Figure 6B shows the regulative role of MECP2 in a network of proteins that involves cell death and survival.

The data from this study indicates that adding 1 *g* on the ISS is effective in reducing the endothelial cell damage and increasing cellular organization and function compared to the µg group. Another study of the same animals used in our present study also showed that 1 *g* AG served to maintain femur bone density and soleus/gastrocnemius muscle mass similar to that in the ground control group, whereas significant decreases in bone density and muscle mass occurred in the flight mice without centrifuge [10]. These studies provide evidence that in-flight AG can mitigate some of the effects of weightlessness during spaceflight. Further investigation will be needed to define the relationship between gravitational dose/time and physiological response by assessing retinal physiological endpoints and function. More rodent centrifugation studies on board the ISS are also needed to provide comprehensive information to compare the effectiveness of the AG prescription in other physiological systems during weightless conditions.

## 4. Material and Methods

### 4.1. Spaceflight and Mouse Condition

Twelve male 9-week old C57BL/6 male mice, obtained from a US breeding colony, were launched 18 July 2016, at the Kennedy Space Center (KSC) on a SpaceX-9 rocket for the 35-day MHU-1 mission to the ISS. The animals were housed in the mouse HCU located in the JAXA “Kibo” facility on the ISS. The 12 flight mice were subdivided into two groups. The first group of flight mice (*n* = 6) were exposed to ambient microgravity conditions (µg group), while the second group of flight mice (*n* = 6) were exposed to continuous artificial Earth gravity (µg + 1 *g* group) while they were in the HCU. AG was achieved through the use of a short-arm centrifuge for the duration of their stay on the ISS. The flight mice were then returned live to Earth and splashed down in the Pacific Ocean on 26 August 2016. It took approximately 40 h for the mice to be recovered in the Pacific Ocean, brought to shore and transported to the testing and processing laboratory located in San Diego, California on 28 August 2016. The spaceflight mice were then euthanized and their eyes were removed and prepared for analysis. Ground control mouse studies were completed in Japan after the return of the flight mice. Control mice (habitat controls, *n* = 6; vivarium controls, *n* = 6) were acquired on 31 August 2016 from a breeding colony in Japan and shipped to the JAXA animal facility in Tsukuba, Japan. HC mice were acclimated to the water lixit system and the same special food bar diet as the space flown mice were fed. They were first housed in the transportation cage unit (TCU) to simulate launch and flight to the ISS, and then placed in the HCUs to simulate the housing conditions experienced by µg mice on the ISS. They were again placed in the TCU to simulate the return to Earth flight. The control mouse dissections took place on 3 November 2016. Control mouse eye tissue was then shipped to the US for analysis. All mice received the same ad libitum access to food and water. A detailed description of the flight schedule and mouse information has been previously reported [11].

The Loma Linda University (LLU) Institutional Animal Care and Use Committee (IACUC) was consulted, but no protocol was required since only archived frozen and fixed tissues were obtained after euthanasia. However, animal experiments were approved by the Institutional Animal Care and Use Committee of University of Tsukuba (No. 16-048), Explora Biolabs (Study Number: EB15-010) on 23 May 2016, JAXA (Protocol Number: 016-014B) on 2 June 2016 and NASA (Protocol Number: NAS-15-004-Y1) on 31 May 2016, and the research with vertebrate animals are done in strict accordance with guidelines and applicable laws in Japan and the United States of America.

### 4.2. Dissecting and Preservation of Mouse Eyes after Spaceflight

Within 2 days of splashing down, mice were euthanized and their eyes were harvested. The posterior half of the right eyecup containing the retina layer, optic disc and a short segment of the optic nerve was placed individually in sterile cryovials, snap frozen in liquid nitrogen and kept at −80 °C prior to use. The left eyes were fixed in 4% paraformaldehyde in phosphate buffered saline (PBS) for immunohistochemistry (IHC) assays.

### 4.3. Immunohistochemistry Assays and Histology

Six µm sections were cut through each eye, and sections were roughly 100 µm apart providing 10 sections per eye for analysis. To characterize apoptosis, 5 sections were subjected to a terminal deoxynucleotidyltransferase dUTP nick-end labeling (TUNEL) staining. Paraffin-embedded sections were deparaffinized in Histo-Clear, then permeabilized in proteinase K solution. Stained sections were evaluated using the DeadEnd™ Fluorometric TUNEL system kit (catalog no. G3250, Promega Corp., Madison, WI, USA). The same sections were then incubated with DyLight 594 *Lycopersicon esculantum*-Lectin (catalog no: DL-11721, Vector Laboratories, Burlingame, CA, USA) at a 1:100 dilution for 30 min at room temperature to stain the endothelium. Nuclei were counterstained with diamidino-2-phenylindole (DAPI, blue). Sections were examined using a BZ-X710 All-in-One inverted fluorescence microscope with structural illumination (Keyence Corp., Elmwood Park, NJ, USA). TUNEL-positive cells were identified by green fluorescence, vascular endothelium were identified with red fluorescence. TUNEL-positive cells that were laid within red lectin-labeled endothelium were identified as TUNEL-positive endothelial cells.

For quantitative analysis, the total number of TUNEL-positive endothelial cells in the retina were determined in 5 sections of each eye. The area of the retina was measured on digital microphotographs using ImageJ counting plugin 1.41 software (National Institutes of Health, Bethesda, MD, USA; http://rsbweb.nih.gov/ij/), and the density profiles were expressed as mean number of apoptotic positive cells/mm^2^. The mean of the density profile measurements across 5 retina sections per eye was used as a single experimental value.

Immunofluorescence staining for an oxidative damage marker on ocular sections was also performed using an anti-4-HNE antibody. Five sections were incubated with the anti-4-HNE antibody (catalog no. HNE11-S, Alpha Diagnostic International Inc., San Antonio, TX, USA) at 4 °C for 2 h followed by a donkey anti-rabbit IgG fluorescence-conjugated secondary antibody (catalog no. A21206, Invitrogen Corp., Waltham, MA, USA) for 2 h at room temperature and counterstained with DAPI.

To determine 4-HNE immunoreactivity, fluorescence intensity was measured on each section and calculated using ImageJ software. Once the green channel was separated from the red channel in an image, fluorescence intensities from the areas of interest were measured using the integral/density feature in the ImageJ program and data were extracted and averaged within the group. Fluorescence was averaged across 5 retinas per group in retinal nuclear layers. Data were normalized with respect to controls. The values were represented as fold changes over controls.

### 4.4. Mass Spectrometry

Protein expression profiles of ocular tissue containing the retina were determined in eighteen mice (*n* = 6/group) from three sample groups: µg, µg + 1 *g*, and HC, using high resolution mass spectrometry.

Five micrograms of protein lysate were resolved by a 4–20% Tris-Glycine SDS-PAGE (Life Technologies Corp., Waltham, MA, USA). The gel was sliced into 24 gel slices that were 2 mm thick for each sample. Gel slices were destained in 50% methanol (Thermo Fisher Scientific, Hampton, NH, USA), 100 mM ammonium bicarbonate (Sigma-Aldrich, St. Louis, MO, USA), followed by reduction in 10 mM Tris (2-carboxyethyl) phosphine (Pierce, Dallas, TX, USA) and alkylation in 50 mM iodoacetamide (Sigma-Aldrich). Gel slices were then dehydrated in acetonitrile (Thermo Fisher Scientific), followed by addition of 100 ng porcine sequencing grade modified trypsin (Promega Corp.) in 100 mM ammonium bicarbonate (Sigma-Aldrich) and incubation at 37 °C for 12–16 h. Peptide products were then acidified in 0.1% formic acid (Pierce). Tryptic peptides were separated by reverse phase Jupiter Proteo resin (Phenomenex, Torrance, CA, USA) on a 100 × 0.075 mm column using a nanoAcquity Ultra Performance LC™ (UPLC™) system (Waters Corp., Milford, MA, USA). Peptides were eluted using a 30 min gradient from 97:3 to 60:40 buffer A:B ratio. (Buffer A = 0.1% formic acid, 0.5% acetonitrile; buffer B = 0.1% formic acid, 90% acetonitrile). Eluted peptides were ionized by electrospray (1.9 kV) followed by MS/MS analysis using collision-induced dissociation on an LTQ Orbitrap Velos mass spectrometer (Thermo Fisher Scientific). MS data were acquired using the Fourier Transform ion cyclotron resonance spectrometry (FTMS) analyzer in profile mode at a resolution of 60,000 over a range of 375 to 1500 *m*/*z*. MS/MS data were acquired for the top 15 peaks from each MS scan using the ion trap analyzer in centroid mode and normal mass range with a normalized collision energy of 35.0.

### 4.5. Data Analysis

Proteins were identified by searching the UniProtKB database (2017-03 release; restricted to Mus musculus; 81,557 entries) using the Andromeda search engine in MaxQuant (version 1.5.7.4, Max Planck Institute of biochemistry, Martinsried, Germany). Search parameters were as follows: trypsin digestion with up to three missed cleavages; fixed modification of carbamidomethyl of cysteine; variable modifications of oxidation on methionine and acetyl on N-terminus; first search set to 5 ppm precursor ion tolerance and the main search was set to 3 ppm; selected label-free quantitation with iBAQ with a minimum ratio of 1. A contaminants database was used for the first search to identify commonly identified contaminants.

The MaxQuant iBAQ values for each sample were converted to relative iBAQ (riBAQ) intensities by dividing the iBAQ intensity for each protein by the sum of all iBAQ intensities for all proteins identified in the sample. The median of all relative iBAQ intensities for each sample was then used to normalize between the samples. The adjusted riBAQ intensities were then imported into Perseus (version 1.5.6.0, Max Planck Institute of biochemistry, Martinsried, Germany). Perseus was used to log_2_ transform the data and impute the missing values using a normal distribution with a width of 0.3 and a downshift of 2 standard deviations. Non-parametric Mann–Whitney U tests were used to compare habitat control with the µg and µg + 1 *g* groups separately. The *p*-values were corrected by Benjamini–Hochberg to account for multiple testing. Fold changes were also calculated for each comparison. Proteins that were significant by the FDR-adjusted *p*-value < 0.05 and that had a fold change greater than 2 were considered to be differentially expressed. The heat maps were generated using the Euclidean distance metric and the data was standardized by the mean and standard deviation before performing hierarchical clustering with pheatmap R package (maintained by Raivo Kolde, Massachusetts General Hospital, Boston, MA, USA). Volcano plots were generated using R. These proteins were then analyzed using the IPA pathway analysis. 

### 4.6. Statistical Analysis

The results obtained from all experiments were analyzed by one-way analysis of variance (ANOVA) followed by Tukey’s post hoc multiple-comparison test (Sigma Plot for Windows, version 13.0; Systat Software, Inc., Point Richmond, CA, USA). The significance level was set at *p* < 0.05. Data are shown as mean ± standard error (SEM).

## 5. Conclusions

The current data demonstrate that spaceflight induces apoptosis in vascular endothelial cells of the retina. Such changes to vasculature endothelial cells could serve to impair barrier function of the BRB and compromise the fidelity of intraocular pressure regulation. Study results also show acute changes in the protein expression profile of space-flown and centrifuged mouse retina. Diverse spaceflight-induced alterations in protein-expression profiles suggest broader changes in eye structure and function. If such alterations occur in the eyes of astronauts, these changes could contribute to acute and sustained modifications in astronauts’ visual acuity. Further studies are needed to elucidate the possible mechanism(s) by which changes in protein-expression profiles are mediated and their structural and functional consequences.

## Figures and Tables

**Table 1:** Differentially expressed proteins identified by IPA in the mouse retina in response to µg vs. habitat control (fold change >2; FDR corrected *p* < 0.05).

Protein Names	Function	Fold-Changes
MECP2	Cell cycle, neurological disorder	5.85
TMEM 109	Cell death and survival, neurological disease	3.69
S100A10	Cell cycle	3.19
HECT D4	Neuronal signaling	2.04
XPO 1	Cell cycle	2.02
CLIP2	Neuronal cell structure and function	−2.96
SOGA3	Glucose metabolism	−2.94
CBFB	Cell repair	−2.83
RAB6A	Cellular assembly	−2.63
PYGB	Immune cell trafficking	−2.58
HSD17B12	Cell death and survival	−2.57
RUVBL2	Cell repair	−2.42
NPLOC4	Cellular organization	−2.29
DAP3	Apoptosis	−2.25
SGCA	Cell cycle	−2.24

**Table 2:** Differentially expressed proteins identified by IPA in the mouse retina in response to µg + 1 *g* vs. habitat control (fold change >2; FDR corrected *p* < 0.05).

Protein Names	Function	Fold-Changes
CAPN3	Molecular transport	7.38
CAVIN2	Vascular permeability	−3.93
EDC4	Lipid metabolism	−2.94
KLC2	Cell death and survival	−2.87
ClIP2	Neuronal cell structure and function	−2.74
MAP4	Lipid metabolism	−2.22

**Table 3:** Differentially expressed proteins identified by IPA in the ocular tissue in response to µg vs. µg + 1 *g* (fold change >2, FDR corrected *p* < 0.05).

Protein Names	Function	Fold-Changes
RPL10	Cell death and survival	4.60
C3	Cell morphology and assembly	4.29
CLASP1	Cell assembly and organization	4.26
HDGFL2	Angiogenesis and neuronal signaling	4.22
RPS27L	Protein synthesis	4.16
THTPA	Protein phosphorylation	3.91
TPM1	Cell structure and function	3.91
NCND	Cell cycle	3.76
RPL23	Protein catabolism	3.69
CNPYS	Cellular metabolism	−6.27
ITGA5	Cell signaling	−5.93
SF3A2	Cell signaling	−5.42
ENOPH1	Cellular structure	−4.60
SMUG1	Cellular organization and repair	−4.02
KYAT3	Cell metabolism	−3.86
FBXO22	Protein catabolism	−3.03
